# Association Between Dietary Fatty Acid Pattern and Risk of Oral Cancer

**DOI:** 10.3389/fnut.2022.864098

**Published:** 2022-05-16

**Authors:** Yi Fan, Yu Qiu, Jing Wang, Qing Chen, Sijie Wang, Yaping Wang, Yanni Li, Yanfeng Weng, Jiawen Qian, Fa Chen, Jing Wang, Bin Shi, Lizhen Pan, Lisong Lin, Baochang He, Fengqiong Liu

**Affiliations:** ^1^Fujian Provincial Key Laboratory of Environment Factors and Cancer, Department of Epidemiology and Health Statistics, School of Public Health, Fujian Medical University, Fuzhou, China; ^2^Key Laboratory of Ministry of Education for Gastrointestinal Cancer, Fujian Key Laboratory of Tumor Microbiology, Fujian Medical University, Fujian, China; ^3^Department of Oral and Maxillofacial Surgery, The First Affiliated Hospital of Fujian Medical University, Fujian, China; ^4^Laboratory Center, School of Public Health, The Major Subject of Environment and Health of Fujian Key Universities, Fujian Medical University, Fujian, China

**Keywords:** fatty acid pattern, saturated fatty acids, oral cancer, principal component analysis, case-control study

## Abstract

**Objective:**

To investigate the association between dietary fatty acid (FA) patterns and the risk of oral cancer.

**Method:**

A case-control study which included 446 patients with oral cancer and 448 controls subjects was conducted in Southeast China. A structured food frequency questionnaire was used to assess the dietary FA consumption before cancer diagnosis. FA patterns were identified using the principal component analysis, and the relationship between the dietary FA patterns and oral cancer was analyzed by logistic regression.

**Results:**

General differences in FA intake were observed between the patient and control groups. The intakes of saturated FAs (SFAs) C14:0, C16:0, C18:0, and monounsaturated FA C18:1 were higher in the patient group than the control group (*p* < 0.001). Four FA patterns were derived by principal component analysis. The “SFA” pattern, “Polyunsaturated FA” pattern, “Monounsaturated FA” pattern, and “Medium- and long-chain FA” pattern, which could explain 75.7% of the variance of the dietary FA intake, were submitted to logistic regression analysis. A positive association was observed between the “SFA” pattern and oral cancer risk. Compared with the lowest quartile score, the *OR* of the highest quartile score was 3.71 (95%*CI*: 2.31, 5.94, *P*_*trend*_ < 0.001) in the multivariate logistic regression model. No significant association was found among the other three patterns and oral cancer risk.

**Conclusions:**

General differences in dietary FA intake were observed between patients with oral cancer and controls. A positive association between the “SFA” pattern and risk of oral cancer was observed after adjusting for potential confounders.

## Introduction

Oral cancer is one of the foremost cancers in head and neck cancers with nearly 40,000 new cases recognized in China in 2015 (1). According to GLOBOCAN 2018, the incidence and mortality of oral cancer in China were 2.0/100,000 and 0.97/100,000, respectively, in 2018 (2). The recognized etiologic factors of oral cancer consist of smoking, drinking, oral hygiene, human papillomavirus (HPV), and betel quid consumption (3–9). In addition to the above-mentioned traditional risk factors, diet is also involved in the etiology of oral cancer (10–12). Additionally, the potential role of fatty acids (FAs) in tumorigenesis has got increased interest.

Fatty acids, including saturated FA (SFA), n-3 and n-6 polyunsaturated FA (PUFA), and trans fatty acid (TFA), have been reported to be associated with the risk of varied types of cancer such as prostate cancer (13, 14), pancreatic cancer (15, 16), colorectal cancer (17, 18), and lung cancer (19). However, reports about the association between FA and head and neck tumors, especially oral cancer, are rare.

Most of the previous studies have taken individual FAs as separate exposures. However, individual FAs were consumed together and tended to be correlated with each other and to be interactive or synergistic, partially due to shared food sources and metabolic pathways (20, 21). Because of the complexity of diet and the highly interrelated nature of dietary exposures, FA pattern analysis could instead offer a more comprehensive view of separate FAs and shed light on the biological interactions between different FAs and their relation with disease risk (22–24).

Due to the limited evidence of the role of FA in oral cancer, we performed a case-control study to explore the potential FA intake patterns in oral cancer and their role in the development of oral cancer.

## Materials and Methods

### Study Design and Population

In this case-control study from September, 2016, to July, 2020, 446 newly diagnosed patients with oral cancer and 448 control participants were recruited from the First Affiliated Hospital of Fujian Medical University in Fujian province, China. Cancers of the lip, oral cavities, and parotid corresponded to codes C00 to C07 according to the 10th revision of the International Classification of Diseases (ICD-10) (25) were referred to as oral cancer in this study. The inclusion criteria of the patients were as follows: (1) histologically confirmed primary oral cancer; (2) Chinese Han population and residence in Fujian Province; (3) age above 18 years old. Patients with second primary, recurrent, or metastasized cancer, and previous radiotherapy or chemotherapy were excluded. Control participants were recruited from the health examination center of the same hospital during the same period. Those with a history of cancer were excluded. Additionally, we excluded those with extreme daily caloric intake (>4,200 or <700 kcal/day for men; >3,500 or <500 kcal/day for women).

All participants provided signed informed consent. The study protocol was approved by the Institutional Review Board of Fujian Medical University (Approval number: 2011053; Approval date: March 10, 2011) and conducted following the ethical standards described in the Declaration of Helsinki.

### Data Collection

A structured questionnaire was used to collect information through face-to-face interviews conducted by well-trained interviewers. The questionnaire included socio-demographic characteristics (age, sex, education, marital status, residence, occupation, and family history of cancers) and lifestyle indicators (tobacco smoking, alcohol drinking, and oral hygiene). Subjects who had smoked at least 100 cigarettes during their lifetime were considered tobacco smokers. Alcohol drinker was defined as consuming at least one drink per week and lasting for more than 6 months continuously (26). A complete description of the oral hygiene score is available in our previous study (3). Oral hygiene score = teeth brushing + the number of missing teeth + wearing dentures + regular dental visits + recurrent dental ulceration. The range of oral hygiene score was 0–8, and a higher score indicated worse oral hygiene. Detailed coding information of variables included in the analysis was as follows: age (<49 years/≥49 years, based on the median of controls), sex (male/female), marital status (married/others), residence (rural areas/urban areas), occupation (farmer and worker/others), tobacco smoking (no/yes), alcohol drinking (no/yes), oral hygiene (0–2/3–5/6–8), and family history of cancer (no/yes). Educational was defined as low (lower vocational training or primary school), or high (secondary school and above) level groups. Height and weight were measured by the nurse of the hospital. The body mass index (BMI) was calculated as weight (in kilograms) divided by the square of the height (in meters) and was classified into three categories (<18.5/18.5–23.9/≥24).

A validated food frequency questionnaire (FFQ) (27) was utilized to collect the habitual dietary intake from each participant. The dietary intake of the year before the interview was collected. The dietary items were grouped into 8 broad categories (grains; beans and soy products; vegetables; fruits; animal food; algal fungi and nuts; beverages and soup; fried foods and pickled foods) and 17 sub-categories (grains; beans and soy products; dark vegetables; light color vegetables; purple vegetables; fresh beans; fruits; livestock; poultry; fish; processed meat; red meat; eggs; dairy; algal fungi and nuts; fried foods; pickled foods). For each food item or food group, participants were asked how frequently (daily, weekly, monthly, yearly, or never) they consumed the food or food group, which was followed by a question on the amount consumed in lians per unit of time. Lian is a unit of weight in China (1 lian = 50 g). The Chinese Food Composition Tables (28) were used to estimate the intake levels of macronutrients and FAs for participants.

### Statistical Analysis

The intakes of energy and nutrients were log transformed and then FA intakes were adjusted for total energy intake using the residuals method (29). The quantitative data were presented as median with inter-quartile range, while the qualitative variables were presented as frequency (numbers and percentages). The chi-square test was used to compare the main characteristics between patients and controls. The Wilcoxon rank-sum test was used to analyze the distribution of dietary FAs. The Pearson correlation coefficients were calculated, and the hierarchical cluster tree and heatmap were generated to visualize the correlation between FAs (30). Hierarchical cluster analysis was performed using the Ward's method on correlation coefficient using the pheatmap package in R software.

Fatty acid patterns were derived by principal component analysis (PCA) using the intake of 32 FAs and PCs identified were referred to as FA patterns. The correlation pattern matrix from PCA was then used to calculate the scores of each pattern which were then categorized into quartiles, and the lowest quartiles were used as reference. The FA pattern score was evaluated categorically in the logistic regression model, and the *OR*s and their 95% *CIs* were calculated. Associations between FA pattern and intakes of 17 food groups and macronutrients were assessed by the Spearman correlation analysis. In addition, the restricted cubic spline (RCS) was used to plot and investigate the possible non-linear association between FA pattern and oral cancer risk.

All analyses were performed using the R software (version 4.0.3), with 2-tailed *p*-values <0.05 considered statistically significant.

## Results

### Characteristics of the Study Population

The distributions of the demographical characteristics and lifestyle factors are shown in Table 1. Compared with the patient group, the case group was characterized by a higher proportion of subjects with tobacco abuse, alcohol consumption, tumor history, and worse oral hygiene. In addition, the distribution of gender, education levels, BMI, and residence was significantly different between the patient and control groups (*p* < 0.05). General differences in FA intake were observed between the patient and control groups. The intake of saturated FAs C14:0, C16:0, C18:0, and monounsaturated FA C18:1 were higher in the patient group than the control group (*p* < 0.001). The distribution of dietary FAs between the case and control groups are shown in Supplementary Figure 1.

**Table 1:** Characteristics of the case (*n* = 446) and control (*n* = 448) group.

**Variable**	**Case**	**Control**	***P***
Age			**<0.001**
<49	94 (21.1%)	210 (46.9%)	
≥49	352 (78.9%)	238 (53.1%)	
Sex			**0.002**
Male	258 (57.8%)	213 (47.5%)	
Female	188 (42.2%)	235 (52.2%)	
Education			**<0.001**
Low	77 (17.3%)	204 (45.5%)	
High	369 (82.7%)	244 (54.5%)	
Marital status			0.699
Married	408 (91.5%)	413 (92.2%)	
Others	38 (8.5%)	35 (7.8%)	
BMI			**0.024**
<18.5	39 (8.7%)	19 (4.2%)	
18.5~	284 (63.7%)	297 (66.3%)	
≥24	123 (27.6%)	132 (29.5%)	
Residence			**0.008**
Rural areas	258 (57.8%)	298 (66.5%)	
Urban areas	188 (42.2%)	150 (33.5%)	
Occupation			0.231
Farmer and worker	148 (33.2%)	132 (295%)	
Others	298 (66.8%)	316 (66.8%)	
Tobacco smoking			**0.001**
No	259 (58.1%)	307 (68.5%)	
Yes	187 (41.9%)	141 (31.5%)	
Alcohol drinking			**<0.001**
No	294 (65.9%)	349 (77.9%)	
Yes	152 (34.1%)	99 (22.1%)	
Family history of tumor			**<0.001**
No	374 (83.9%)	413 (92.2%)	
Yes	72 (16.1%)	35 (7.8%)	
Oral hygiene score			**<0.001**
0–2	79 (17.7%)	167 (37.3%)	
3–5	257 (57.6%)	248 (55.4%)	
6–8	110 (24.7%)	33 (7.4%)	

### Identification of FA Patterns

**Figure 1:**
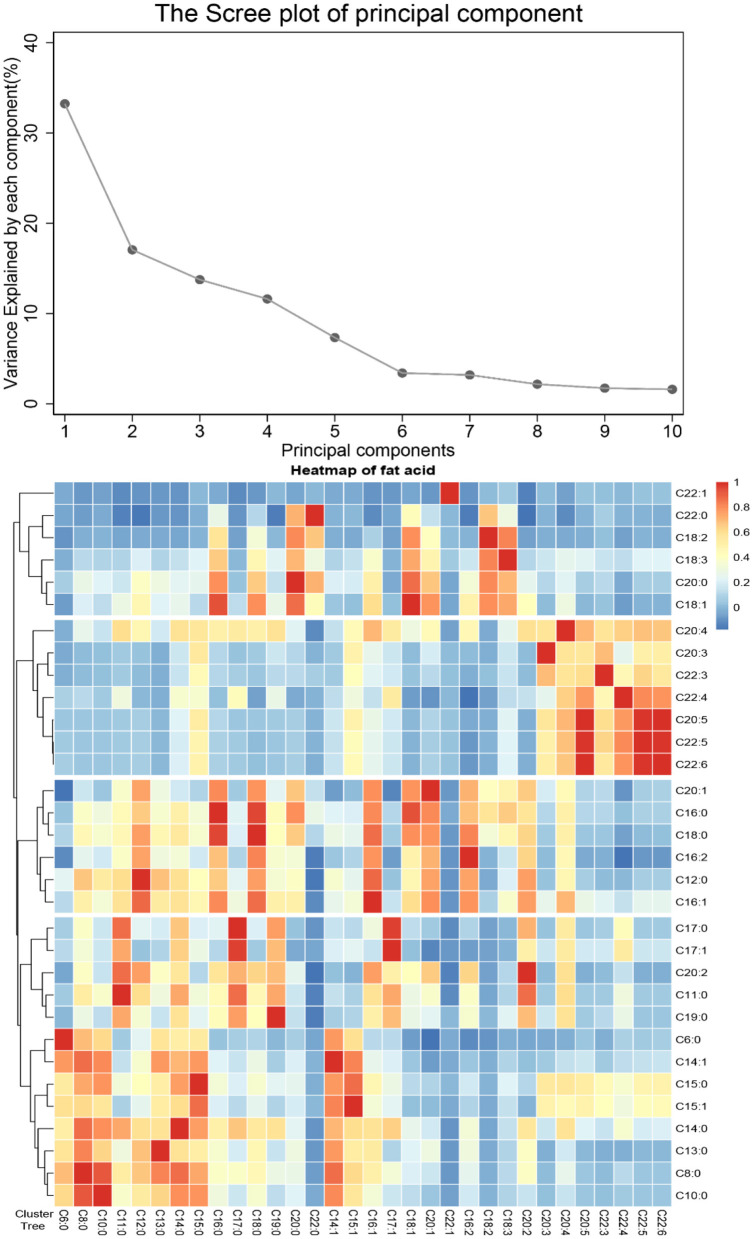
Principal components and clusters of 32 fatty acids. **(A)** The proportion of total variance of 32 fatty acids explained by each principal component. **(B)** Association among 32 fatty acids, the hierarchical cluster tree on the left, and the heatmap of fatty acid on the right.

Four FA patterns were identified by applying PCA which could explain 75.7% of the variance of the dietary FA consumption, as the scree plot shown in Figure 1. Pattern 1 was characterized with saturated FA (the “SFA” pattern), which mainly included octanoic acid (C8:0), undecanoic acid (C11:0), lauric acid (C12:0), myristic acid (C14:0), and pentadecanoic acid (C15:0). Pattern 2 (the “PUFA” pattern) had high factor loading of eicosatrienoic acid (C20:3), eicosapentaenoic acid (C20:5), docosatrienoic acid (C22:3), docosatetraenoic acid (C22:4), docosapentaenoic acid (C22:5), and docosahexaenoic acid (C22:6). Pattern 3 (the “MUFA” pattern) was characterized with oleic acid (C18:1), eicosenoic acid (C20:1), and erucic acid (C22:1). Pattern 4 [the “medium- and long-chain FA (MLC-FA)” pattern] was dominated by heptadecanoic acid (C17:0), behenic acid (C20:0), myristoleic acid (C14:1), heptadecenoic acid (C17:1), and eicosadicnoic acid (C20:2). The factor loadings of individual FAs in the four FA patterns are shown in Table 2. Additionally, a correlation analysis among individual FAs was performed, and a heatmap was derived using correlation coefficients among individual FAs. A similar pattern was identified in the cluster analysis, as FAs adjacent in the tree had similar loading values (Figure 1).

**Table 2:** Factor-loading matrix for four fatty acid patterns.

**Fatty acids**	**Name**	Fatty acid patterns^*^			
		**“SFA” pattern**	**“PUFA” pattern**	**“MUFA” pattern**	**“MLC-FA” pattern**
**Saturated fatty acids**
C6:0	Caproic			−0.538	
C8:0	Caprylic	**0.731**			
C10:0	Capric	**0.596**			
C11:0	Undecanoic	**0.702**			
C12:0	Lauric	**0.783**			
C13:0	Tridecanoic	**0.618**			
C14:0	Myristic	**0.848**			
C15:0	Pentadecanoic	**0.720**			
C16:0	Palmitic	**0.787**			
C17:0	Heptadecanoic				**−0.642**
C18:0	Stearic	**0.792**			
C19:0	Non-adecanoic	**0.586**			
C20:0	Arachidic	**0.559**			
C22:0	Behenic				**0.410**
**Monounsaturated fatty acids**
C14:1	Myristoleic				**0.519**
C15:1	Pentadecanoic			−0.132	
C16:1	Palmitoleic	0.871			
C17:1	Heptadecenoic				**−0.549**
C18:1	Oleic			**0.355**	
C20:1	Eicosenoic			**0.337**	
C22:1	Erucic			**0.088**	
**Polyunsaturated fatty acids**
C16:2	Hexadecatrienoic	0.577			
C18:2	Linoleic		−0.486		
C18:3	Octadecadienoic			0.477	
C20:2	Eicosadienoic	0.695			**−0.592**
C20:3	Eicosatrienoic		**0.433**		
C20:4	Arachidonic	0.769			
C20:5	Eicosapentaenoic		**0.658**		
C22:3	Docosatrienoic		**0.474**		
C22:4	Docosatetraenoic		**0.651**		
C22:5	Docosapentaenoic		**0.658**		
C22:6	Docosahexaenoic		**0.654**		

* *Four principal components explained 75.7% of the variation in all 32 fatty acids*.

### Association Between FA Patterns and Oral Cancer Risk

Crude and multivariable-adjusted *OR* and 95% *CI* for oral cancer across quartile categories of dietary FA pattern scores are shown in Table 3. A positive association between the “SFA” pattern and the risk of oral cancer was observed. In the crude model, those in the highest quartile of the “SFA” pattern had an increased risk of oral cancer compared with the lowest quartile, with a statistically significant linear trend (*OR* = 3.36; 95% *CI*: 2.28–4.96; *P*_*trend*_ < 0.001). In model 1, after adjusting for sex, age, marital status, education levels, residence, BMI, occupation, and family history of tumor, the individuals in the highest quartile of the “SFA” pattern tended to have higher oral cancer risk (*OR* = 3.00; 95%*CI*: 1.93–4.68; *P*_*trend*_ < 0.001) compared with those in the lowest quartile. In model 2, the result remained statistically significant after further adjustment for lifestyle factors, including tobacco smoking, alcohol drinking, and oral hygiene score (*OR* = 3.71; 95% *CI*: 2.31–5.94; *P*_*trend*_ < 0.001). Compared with the lowest quartile, the *OR*s of the second quartile of the “PUFA” pattern were 0.58 (95% *CI*: 0.38–0.89) and 0.55 (95% *CI*: 0.35–0.85) in the crude model and model 2. Additionally, the *OR*s of the highest quartile of the “PUFA” pattern were 1.79 (95% *CI*: 1.23–2.62) and 1.59 (95% *CI*: 1.04–2.44) compared to the lowest quartile in the crude model and model 1. However, the result showed no statistical significance after further adjustment in model 3 (*OR* = 1.38; 95% *CI*: 0.88–2.16). Neither the “MUFA” nor the “MLC-FA” pattern was observed to be associated with oral cancer in all the three models (*P* > 0.05).

**Table 3:** Association between fatty acid patterns and oral cancer risk.

**Model^**#**^**	Quartiles of the fatty acid pattern score^*^				***P_***trend***_***
	**I**	**II**	**III**	**IV**	
**“SFA” pattern**
Case/control (*n*)	138/86	139/84	99/125	72/151	
Crude	1.0 (reference)	0.97 (0.66, 1.42)	2.06 (1.39, 2.95)	3.36 (2.28, 4.96)	<0.001
Model 1	1.0 (reference)	0.93 (0.60, 1.43)	2.24 (1.46, 3.44)	3.00 (1.93, 4.68)	<0.001
Model 2	1.0 (reference)	1.07 (0.68–1.68)	2.56 (1.62, 4.02)	3.71 (2.31, 5.94)	<0.001
**“PUFA” pattern**
Case/control (*n*)	116/107	137/87	111/113	84/139	
Crude	1.0 (reference)	0.68 (0.47, 1.00)	1.10 (0.76, 1.59)	1.79 (1.23.2.62)	<0.001
Model 1	1.0 (reference)	0.58 (0.38, 0.89)	0.99 (0.65, 1.15)	1.59 (1.04, 2.44)	0.006
Model 2	1.0 (reference)	0.55 (0.35, 0.85)	0.92 (0.59, 1.42)	1.38 (0.88, 2.16)	0.038
**“MUFA” pattern**
Case/control (*n*)	101/123	125/98	119/105	103/120	
Crude	1.0 (reference)	0.64 (0.44, 0.94)	0.73 (0.50, 1.05)	0.96 (0.66, 1.39)	0.980
Model 1	1.0 (reference)	0.67 (0.44, 1.03)	0.75 (0.49, 1.14)	1.03 (0.67, 1.56)	0.762
Model 2	1.0 (reference)	0.68 (0.44, 1.06)	0.78 (0.50, 1.20)	1.15 (0.74, 1.78)	0.441
**“MLC-FA” pattern**
Case/control (*n*)	111/113	117/106	123/101	97/126	
Crude	1.0 (reference)	0.89 (0.61, 1.29)	0.81 (0.56, 1.17)	1.28 (0.88, 1.85)	0.290
Model 1	1.0 (reference)	0.69 (0.45, 1.05)	0.69 (0.45, 1.06)	0.99 (0.65, 1.52)	0.993
Model 2	1.0 (reference)	0.72 (0.46, 1.12)	0.69 (0.45, 1.08)	1.02 (0.66, 1.58)	0.928

* *Four categories were obtained by quartiles of the fatty acid pattern scores. Each participant was assigned a fatty acid pattern score for each pattern*.

# *Mode l adjusted for demographic characteristics including sex, age, marital status, residence, BMI, family history of tumor, occupation, education*.

*Model 2 adjusted for demographic characteristics and tobacco smoking, drinking, oral hygiene score*.

Additionally, we evaluated the correlations between the “SFA” pattern with intakes of macronutrients and food groups, the result of which is shown in Supplement Table 1. The “SFA” pattern was positively associated with the intake of protein, total fat (r = 0.207, 0.368, respectively, all *p* < 0.001), but negatively related to fiber (r = −0.185, *P* < 0.001). As for food groups, the “SFA” pattern was positively correlated with the intakes of fish, eggs, dairy, and red meat (r = 0.372, 0.320, 0.283, 0.282, respectively, all *p* < 0.05), but negatively correlated with grain and vegetables (r = −0.403, −0.100, respectively, all *p* < 0.05).

**Figure 2:**
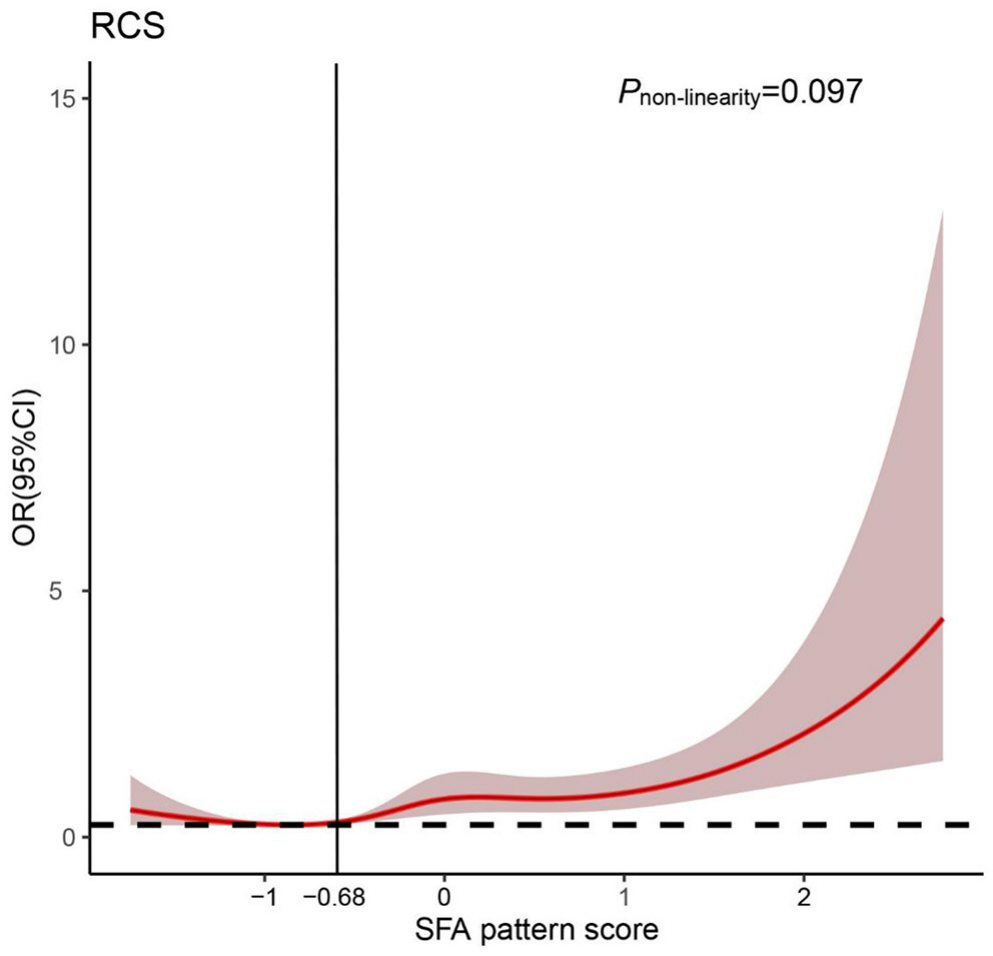
Odds ratio (*OR*) based on saturated fatty acid “(SFA) pattern” score and restricted cubic spline (*P*_non−linearity_ = 0.097). OR was adjusted for the same variates as in model 2.

Furthermore, we visualized the association between the “SFA” pattern score and the risk of oral cancer using restricted cubic splines. Generally, the risk of oral cancer increased with the increase of the “SFA” pattern score and there is no evidence of non-linear association between the score and oral cancer risk (*P*_non−linearity_ = 0.097). However, the risk of oral cancer was relatively flat until around −0.68 of the “SFA” pattern scores and then started to increase rapidly afterward (Figure 2).

### Association Between “SFA” Pattern and Oral Cancer Risk by Stratification Analysis

**Figure 3:**
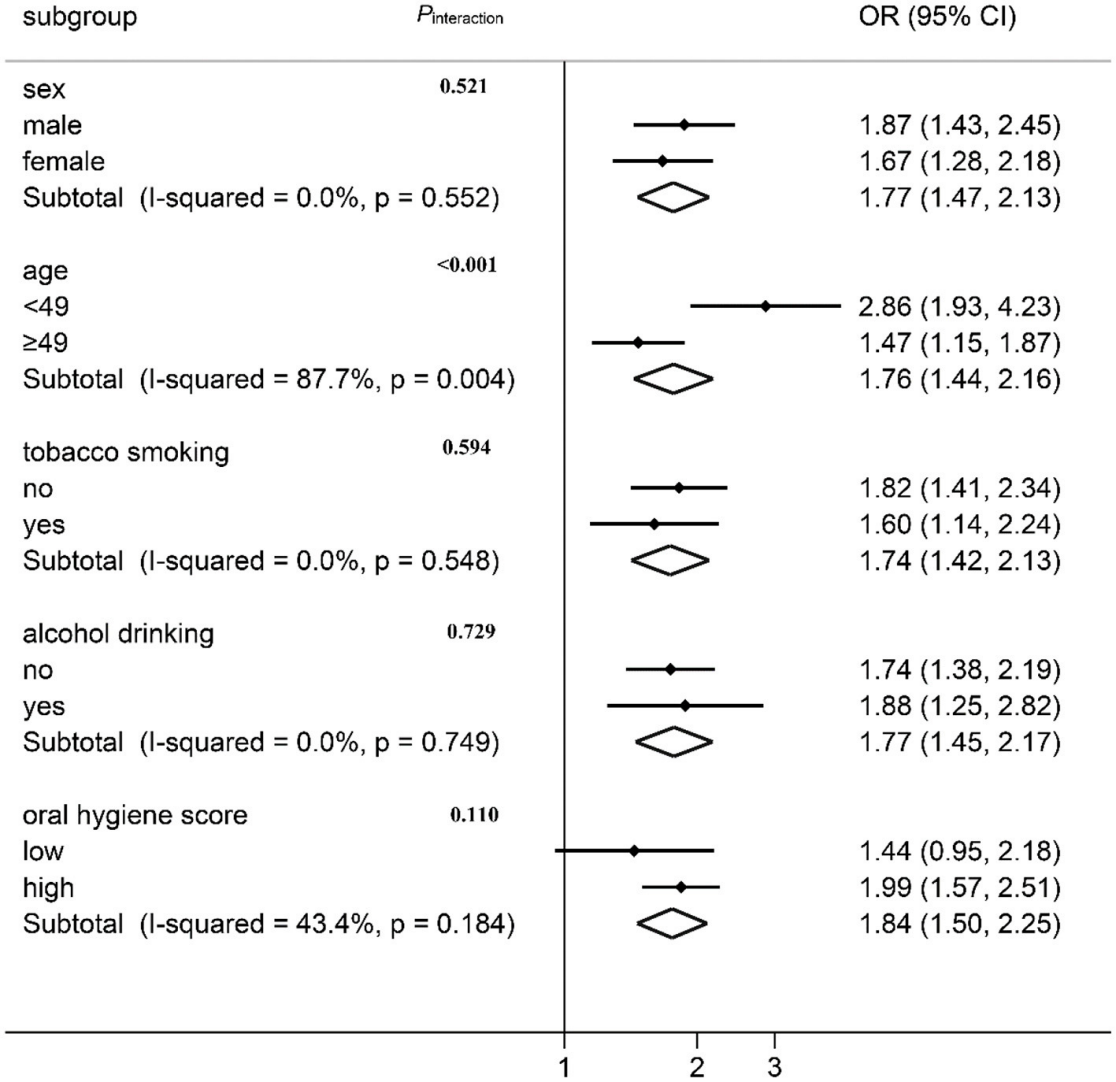
Association between “SFA” pattern and oral cancer risk by stratification analysis.

The association between the “SFA” pattern and oral cancer risk was stratified by the demographic characteristics and lifestyle factors, the result of which is shown in Figure 3. A positive association between oral cancer risk and the “SFA” pattern was observed in all subgroups except for the lower oral hygiene score group. No effect modification was observed by sex, tobacco smoking, alcohol drinking, or oral hygiene score (*P*_*heterogeneity*_ > 0.05). The association varied across different age groups (Figure 3; *I*^2^ = 87.8%, *P*_*heterogeneity*_ = 0.004). The interaction was further tested by multiplying the variates of “SFA” pattern score with age in the logistic regression model, and a multiplicative interaction was observed (*P*_*interaction*_ < 0.001).

## Discussion

In this case-control study conducted in Southeast China, we observed that the intake of FAs varied between patients with oral cancer and healthy controls. Four FA patterns, the “SFA” pattern, “PUFA” pattern, “MUFA” pattern, and “MLC-FA” pattern, were derived by PCA. The “SFA” pattern was found to be positively associated with oral cancer risk while no statistically significant association was found between the other three patterns and disease risk.

Dietary FAs, especially saturated FAs, have been hypothesized to increase cancer risk. Kim et al. performed a cross-sectional study, in which the results showed that the risk of colorectal cancer increased with higher SFA intake in Korean adults (31). Several epidemiological studies discovered that increased consumption of SFA correlated with increased odds of prostate cancer and may also be directly associated with the risk of biochemical recurrence and cancer progression (30, 32, 33). However, there is also evidence supporting that dietary SFA is not associated with cancer risk or even negatively associated with cancer risk. Cao et al. performed a meta-analysis of prospective cohort studies, in which the results showed that the highest vs. lowest levels of dietary SFA were not associated with the risk of breast cancer (34). A meta-analysis of prospective cohort research shows a null association between the SFA intake and colon cancer risk (35). No associations were observed in the Nurses' Health Study cohort of dietary SFAs and epithelial ovarian cancer risk (36). In the European Prospective Investigation into Cancer and Nutrition (EPIC), Aglago et al. (17) found an inverse association between dietary total SFA and colorectal cancer. To the best of our knowledge, reports of the association between dietary SFA and oral cancer are rare. A FA pattern characterized by SFA was identified in our study and was found to be positively associated with oral cancer risk. The inconsistent findings across studies may be partly due to differences in the type of cancer, study design and population, sample size, and varied measuring of dietary intake and confounding elimination.

The mechanism concerning dietary SFA and risk of cancer had also been widely discussed. It was reported that SFA intake influenced the risk of oral cancer through several mechanisms including chronic inflammation, insulin resistance, and fatty acylation, which were all related to carcinogenesis. Firstly, dietary SFA, particularly lauric acid and palmitic acid, were capable of stimulating inflammatory response through the toll-like receptors 4 (TLR4) (37), which could be exacerbated by the production of reactive oxygen species (ROS) *in vivo* (38). Inflammation was a key cause of the development and progression of many chronic diseases, including cancer (39). Moreover, inflammatory cytokines such as tumor necrosis factor (TNF)-a, induced by SFA, may influence insulin sensitivity (40), which favored the establishment of a pro-tumorigenic environment (41). Fatty acylation was another potential carcinogenic mechanism of SFA. It was shown that an SFA-rich diet could lead to an increase of myristoylated Src kinase and Src-mediated oncogenic signaling which accelerated tumor progression (42).

Dietary intake of SFAs consists of both animal and plant origins. The association between dietary FAs and cancer risk may depend on types and food sources of FAs (43, 44). The “SFA” pattern identified in this study was verified by performing a correlation analysis between the “SFA” pattern score and intakes of nutrients and food groups. It was found that the intake of red meat and dairy products was significantly higher in individuals with higher “SFA” pattern scores, which was consistent with previous studies about relation between varied food components and oral cancer. A study from Italy suggested that animal-derived foods such as dairy products and red meat could increase the risk of oral cancer (45). Epidemiological evidence from Greece also indicated that meat products were positively associated with the risk of oral cancer (46). However, we did not observe significant food components of plant origin that were related to SFA intake. So, it was unclear whether the association between the “SFA” pattern and oral cancer was partially attributed to the origin of SFA intake. This remains unclear for now and warrants investigation.

Additionally, in stratification analysis, we found that the association between the “SFA” pattern and oral cancer risk varied with age. The “SFA” pattern was positively associated with oral cancer risk in both age groups, but the association was more significant in the age group younger than 49 years. Compared with MUFA and PUFA, SFA is more likely to come from red meat, processed meat, and dairy products. Red meat is a primary source of total SFA, which has been identified as a dietary risk factor closely associated with various cancers (47, 48). In addition to red meat, excessive intake of dairy products could also contribute to cancer risks (49). Therefore, the origin of SFAs may modulate the effect of SFAs on oral cancer risk. Actually, in this study, we found that the “SFA” pattern was more strongly associated with dairy products in the younger-age group than the older-age group (Supplement Table 2). The results indicate that younger-age groups may consume more saturated FAs from dairy products, such as cakes, cheese, and ice cream bars, which may be positively associated with the risk of oral cancer.

There were several limitations in this study. Firstly, the selection of controls was not well-matched with the case, which resulted in distribution differences between the case and control groups in characteristics such as sex, age, and education. This could imply a selection bias, even when these variables were adjusted in the models. Secondly, recall bias and measurement error in dietary assessment using FFQ could be hardly avoided in a case-control study. Lastly, this was a single-center study and the sample size was limited. A prospective study with a large-scale sample size is needed to verify the current findings

## Conclusion

In conclusion, the study provides support for a possible positive relationship between the “SFA” pattern and the risk of oral cancer. In addition, potential interactions were found between “SFA” pattern and age in oral cancer risk. Our findings support previous findings that there is suggestive evidence of a link between dietary patterns with head and neck cancer, but go beyond this by highlighting the role of specific FA patterns in oral cancer susceptibility.

## Data Availability Statement

The original contributions presented in the study are included in the article/Supplementary Materials, further inquiries can be directed to the corresponding author/s.

## Ethics Statement

The studies involving human participants were reviewed and approved by the Institutional Review Board (IRB) of Fujian Medical University. The patients/participants provided their written informed consent to participate in this study.

## Author Contributions

YF, JW, QC, and FL conceptualized the original idea for the study and have been involved in data collection, data analysis, and manuscript drafting. YQ, LL, LP, and BS were involved in data and blood samples collection. SW, YWa, YL, YWe, and JQ carried out the initial analysis. FC and BH assisted with revisions. All authors have made substantial contributions to the conception and design of the study, read and approved the final manuscript, and agreed to be accountable for all aspects of the work.

## Funding

This work was supported by the Fujian Natural Science Foundation Program (grant number: 2020J01639), Technology Development Fund from the Department of Education of Fujian Province (grant number: 2019L3006), and Fujian Provincial Health Technology Project (No. 2018-1-57).

## Conflict of Interest

The authors declare that the research was conducted in the absence of any commercial or financial relationships that could be construed as a potential conflict of interest.

## Publisher's Note

All claims expressed in this article are solely those of the authors and do not necessarily represent those of their affiliated organizations, or those of the publisher, the editors and the reviewers. Any product that may be evaluated in this article, or claim that may be made by its manufacturer, is not guaranteed or endorsed by the publisher.
