# Supplementary material for: Association Between Dietary Fatty Acid Pattern and Risk of Oral Cancer
Source: Front Nutr. 2022 May 16;9:864098. doi: 10.3389/fnut.2022.864098 (PMC9149618; doi:10.3389/fnut.2022.864098)
Supplement: Supplementary file 1 [file Data_Sheet_1.docx]

**Supplement Figure 1** Distribution of dietary fatty acid intake in case group and control group


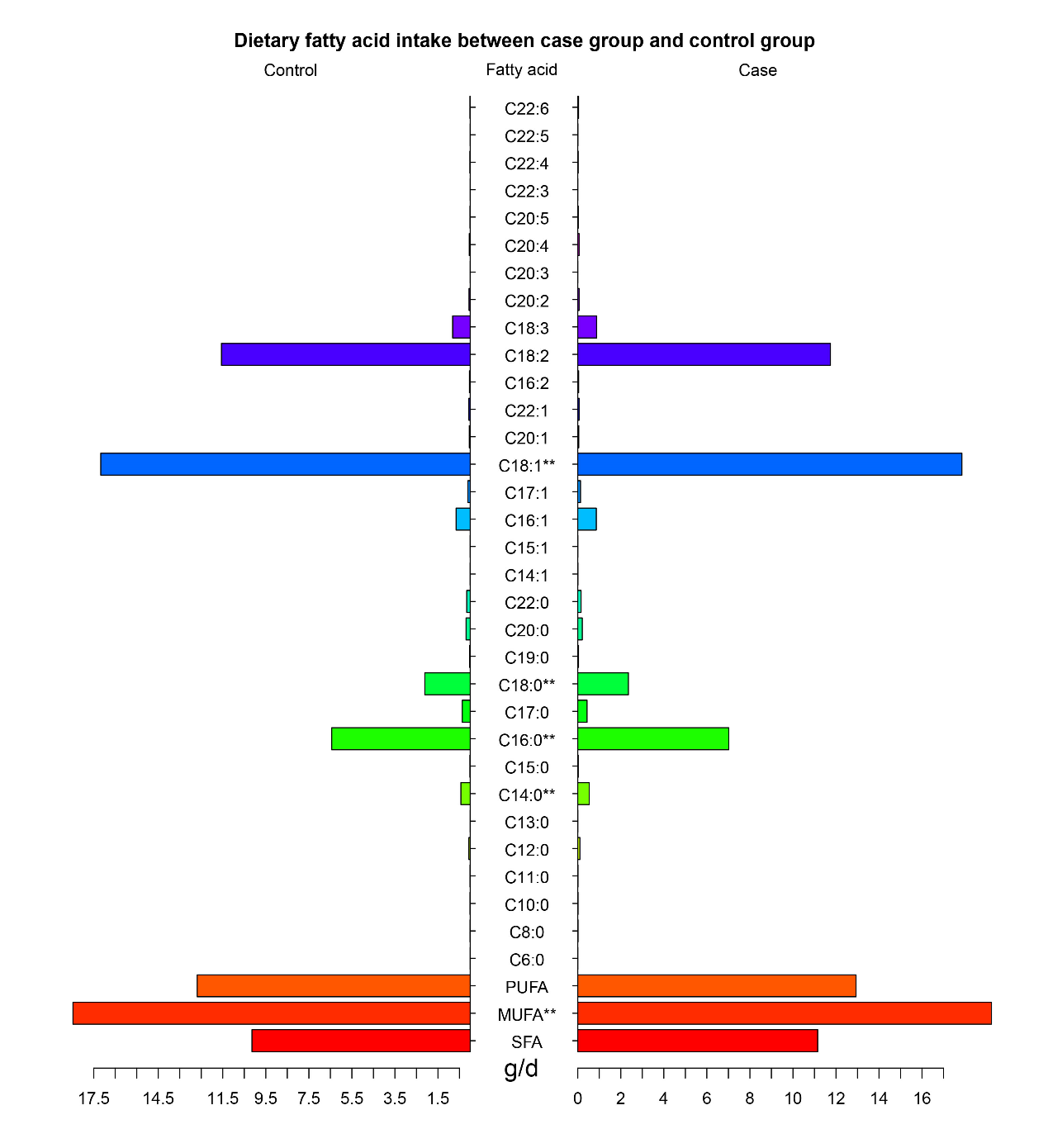


**Supplement Table 1** Spearman correlation coefficients between “SFA” pattern score and the intakes of macronutrients, and food groups in study subjects

|  | “SFA” pattern score | | |
| --- | --- | --- | --- |
|  | Item | r^1^ | *P* |
| Maronutrients^2^ | Protein | 0.207 | ＜0.001 |
|  | Fat | 0.368 | ＜0.001 |
|  | Fiber | -0.185 | ＜0.001 |
| Food groups^3^ | Grain | -0.403 | ＜0.001 |
|  | Vegetables | -0.100 | 0.003 |
|  | Red meat | 0.282 | ＜0.001 |
|  | Fish | 0.372 | ＜0.001 |
|  | Eggs | 0.320 | ＜0.001 |
|  | Dairy | 0.283 | ＜0.001 |

^1^spearman’s correlation coefficients; ^2^All correlation coefficients are significant, all *p*<0.001; ^3^All listed correlations are significant

**Supplement Table 2** Spearman correlation coefficients between “SFA” pattern score and the intakes of food groups stratified by age group

| food group | ＜49(year) | |  | ≥49(year) | |
| --- | --- | --- | --- | --- | --- |
|  | r | P |  | r | P |
| Red meat | 0.266 | ＜0.001 |  | 0.298 | ＜0.001 |
| Fish | 0.403 | ＜0.001 |  | 0.359 | ＜0.001 |
| Dairy | 0.326 | ＜0.001 |  | 0.265 | ＜0.001 |
| Grains | -0.541 | ＜0.001 |  | -0.339 | ＜0.001 |
| Eggs | 0.209 | ＜0.001 |  | 0.377 | ＜0.001 |
